# IL-10 Polymorphism and Breast Cancer Risk in Georgian Women: A Case–Control Study

**DOI:** 10.1055/s-0043-1770957

**Published:** 2023-07-10

**Authors:** Saba Ahmadi, Sandro Surmava, Eka Kvaratskhelia, Elene Abzianidze, Ketevani Kankava

**Affiliations:** 1 Department of Molecular and Medical Genetics, Tbilisi State Medical University, Tbilisi, Georgia

**Keywords:** interleukin-10, single-nucleotide polymorphism, breast cancer, RT-PCR, proliferative activity

## Abstract

**Background** Interleukin-10 (IL-10) is a cytokine with a vast variety of functions, but its role in cancer development and progression is not yet clear. It is involved in two of the hallmarks of cancer: vascularization and immune modulation. IL-10 inhibits angiogenesis and hence is antitumorigenic. But it also can suppress the immune system and be tumorigenic.

**Objective** Evaluating the role of IL-10 (-1082 A/G) gene promoter single-nucleotide polymorphism (SNP) in breast cancer susceptibility and progression in Georgian women.

**Methods** A case–control study was performed on a total of 128 women, with 64 of them being histologically confirmed to have breast cancer and 64 healthy controls. SNP genotyping was performed with TaqMan assay with real-time polymerase chain reaction. And pathology report, containing proliferative activity and breast cancer hormonal status, was obtained after surgery of the case individuals. Statistical analysis was done to investigate the significance of data obtained from genotyping and histology reports.

**Results** Statistical analysis revealed that the difference in frequency of genotypes was not statistically significant between cases and controls (chi-square = 0.5812, *p* = 0.7478). The comparison of proliferative activity of cases with AA genotypes and AG/GG genotypes showed no statistical difference ( *t* = 0.2575, *p* = 0.7980). Although when put into a plot (box and whiskers), patients with AG/GG genotype have outliers with very high proliferative activity.

**Conclusion** This study shows that -1082 A/G SNP in the promoter region of the IL-10 gene is not associated with breast cancer risk in Georgian women.

## Introduction

Breast cancer is the most frequently diagnosed cancer among women and is the second leading cause of cancer mortality in females. In 2019, breast cancer accounted for 30% of estimated new cancer cases in the United States. 1

Today, 5 to 10% of all breast cancers are caused by known germline mutations. These genes are divided into high-risk and low- to moderate-risk susceptibility groups. Genes like *BRCA1* , *BRCA2* , *PTEN* , *TP53* , *LKB1/STK11* , and *CDH1* are among high-risk susceptibility genes, while *CHEK2* , *TGF* β *1* , *CASP8* , and *ATM* are among low- to moderate-risk group. Currently, ongoing case–control studies are dedicated to finding more of such genes. 2 Similarly, this study aims to investigate the role of 1082 single-nucleotide polymorphism (SNP) in the promoter region of interleukin-10 (IL-10) which is known to influence its expression.

Even though the human immune system has advanced ways of averting cancer development, neoplastic cells employ multiple strategies to evade immune detection. One such strategy is producing immunosuppressive factors that dampen the function of T cells, dendritic cells, and natural killer cells. Nitric oxide (NO), IL-6, IL-10, tumor growth factor-beta, indoleamine 2,3-dioxygenase, arginase-1, prostaglandin E2, vascular endothelial growth factor, and cyclooxygenase-2 are all examples of such factors that help cancers evade immunity. Among these factors is IL-10 which can impede the function of CD4+ and CD8+ T cells and promote immunosuppressive cells like Treg 3 ; hence, promoting cancer development and progression. As an example, it was shown that in the case of infection with high-risk human papillomavirus strains, the immunosuppressive properties of IL-10 played a role in facilitating the evasion of immune response by the pathogen, leading to more progressive cervical disease. 4 5

However, the role of IL-10 in cancer is not so straightforward. IL-10 expression has also been linked to the downregulation of major histocompatibility complex class I expression on the cell surface. This change enhances the natural killer cells' detection of tumor targets, leading to the lysis of tumor cells. 6 Also, a study on the effect of IL-10 on melanoma showed that IL-10 can inhibit macrophage-derived angiogenic factors and probably act as an antimetastatic agent. 7

Due to its conflicting immunologic effects, the role of IL-10 in cancer remains uncertain. IL-10 gene is located on chromosome 1 (1q31-1q32), and SNPs in its promoter region, -1082 A/G, can alter its expression and therefore can lead to alterations in cancer susceptibility and cancer progression. 8 9

In this case–control study, we aim to investigate the involvement of this SNP in breast cancer pathogeneses and its clinical manifestations in the Georgian women population.

## Materials and Methods

The study is approved by the Ethics Committee of Tbilisi State Medical University, Tbilisi, Georgia. Signed informed consent was collected from every study participant. The study design is a case–control study. Breast cancer patients who were consecutively admitted to the surgery department in two different oncology hospitals (The First University Clinic and Cancer Research Center) in Tbilisi, Georgia, were recruited from 2017 to 2019. Age-matched women who were regularly involved in breast cancer screening in an outpatient clinic in Tbilisi, and were healthy, were asked to volunteer as control group members. Clinical information of patients was collected from medical notes.

The eligibility criteria for the cases were:

1. Biopsy-confirmed diagnosis of breast carcinoma, with no previous history of breast surgery, and no preoperative chemotherapy or radiation therapy.
2. The age range of 30 to 80 years.
3. Ability to understand the purpose of the study and provide informed consent.
4. Female sex.
5. Being ethnically Georgian.

Eligibility criteria for controls were as followed: No history of previous cancer diagnosis, no family history of cancer, and minor illnesses were acceptable (e.g., common cold, headache). The rest of the criteria (2–5) are identical to that of the cases.

Due to incomplete data, inappropriate material, or nonmatching diagnosis, 11 patients were later excluded from the study. In total, 64 patients were involved in the study along with 64 healthy controls.

### Sample Collection and Storage

Blood samples were collected in a vacutainer tube containing ethylenediaminetetraacetic acid. Genomic deoxyribonucleic acid (DNA) was extracted from the whole blood using DNA purification kits (Qiagen, United States). DNA concentration was measured using the fluorometer-based method (Qubit, Thermo Scientific, United States).

### Genotyping

SNP genotyping was performed using the TaqMan assay (Thermo Scientific). Each TaqMan SNP genotyping assay contained sequence-specific forward and reverse primers to amplify the polymorphic sequence of interest and two TaqMan minor groove binder probes with nonfluorescent quenchers: one VIC-labeled probe to detect Allele 1(A) sequence and one FAM-labeled probe to detect Allele 2(G) sequence.

Real-time polymerase chain reaction (PCR) was performed based on standard protocols. The final volume of PCR reaction was 25 µL, which contained DNA polymerase, forward and reverse primers with a final concentration of 200 nM, probes with a final concentration of 250 nM, and 50 ng of genomic DNA. PCR conditions for amplification included polymerase activation at 95°C for 10 minutes (hold), denaturation at 95°C for 15 seconds, and annealing/extension at 60°C for 1 minute (cycle 40). The real-time PCR instrument software plot the results of the allelic discrimination (AD) data as a plot of Allele 1 (VIC dye) versus Allele 2 (FAM dye). The AD plot represents each sample well as an individual point on the plot. A typical AD plot shows homozygote clusters, a heterozygote cluster, and no-template controls. The points in each cluster are grouped closely together and each cluster is located well away from the other clusters.

### Statistics

The study and control groups were analyzed separately. All statistical analyses were performed by GraphPad Prism 9.3.1 for macOS (GraphPad Software, San Diego, California, United States).

Statistical significance for differences in genotype frequencies was determined by chi-square and Fisher's exact test, and the level of significance was put at *p* < 0.05.

An unpaired *t* -test was used to compare the difference in mean proliferative activity of patients with AA genotype and patients having the G allele (AG and GG).

To evaluate associations between the SNPs and the risk of cancer, odds ratios (ORs) and 95% confidence intervals (CIs) were calculated using unconditional logistic regression analysis. All statistical tests are planned to be two-sided.

## Results

**Fig. 1:**
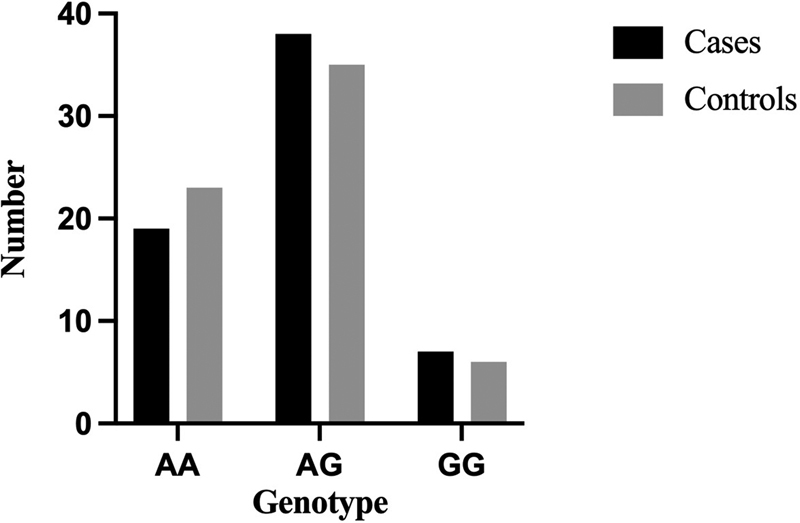
Frequency of each genotype in cases and controls.

Genotyping of IL-10 SNP -1082 A/G was compared between 64 breast cancer cases with 64 healthy controls ( Table 1 ). In the case group, the frequencies of AA, AG, and GG genotypes were 29.68, 59.37, and 10.93%, and in healthy controls 35.93, 54.68, and 9.37%, respectively. The difference in frequency of genotypes was not statistically significant between the two study groups ( Table 1 , chi-square = 0.5812, *p* = 0.7478). Neither IL-10 1082 AA (OR = 0.708, 95% CI = 0.213–2.47) nor IL-10 1082 AG (OR = 0.930, 95% CI = 0.266–2.883) were associated with breast cancer. Overall, there was no association between breast cancer risk and the IL-10 -1082 A/G genotype ( Fig. 1 ).

**Table 1:** Distribution of IL-10 -1082 A/G genotype frequency in breast cancer patients and controls

	Cases, *N* (%)	Controls, *N* (%)	OR (95% CI)	Chi-square	*p* -Value
AA	19 (29.688)	23 (35.938)	0.708 (0.213–2.47)		
AG	38 (59.375)	35 (54.688)	0.930 (0.266–2.883)		
GG	7 (10.938)	6 (9.375)	1.00 Ref	0.5812	0.7478
A allele	76 (59.375)	81 (63.281)	0.848 (0.514–1.390)		
G allele	52 (40.625)	47 (36.718)	1.00 Ref	0.4102	0.5219

Abbreviations: CI, confidence interval; IL-10, interleukin; OR, odds ratio.

Note: The odds ratio of AG/GG to AA is 1.329.

The observed genotype frequencies were in accordance with the Hardy–Weinberg equilibrium.

Among 64 genotyped breast cancer patients, we received pathology reports of 45 patients. The proliferative activity, estrogen receptor status, progesterone receptor status, and Her2/neu status were reported and their frequency is shown in Table 2 .

**Table 2:** Breast cancer status of patients

Category	Frequency (%)
Proliferative activity ⩾ 30	31.11
Proliferative activity < 30	68.88
Her2+	17.77
ER+	71.11
PR+	68.88

**Fig. 2:**
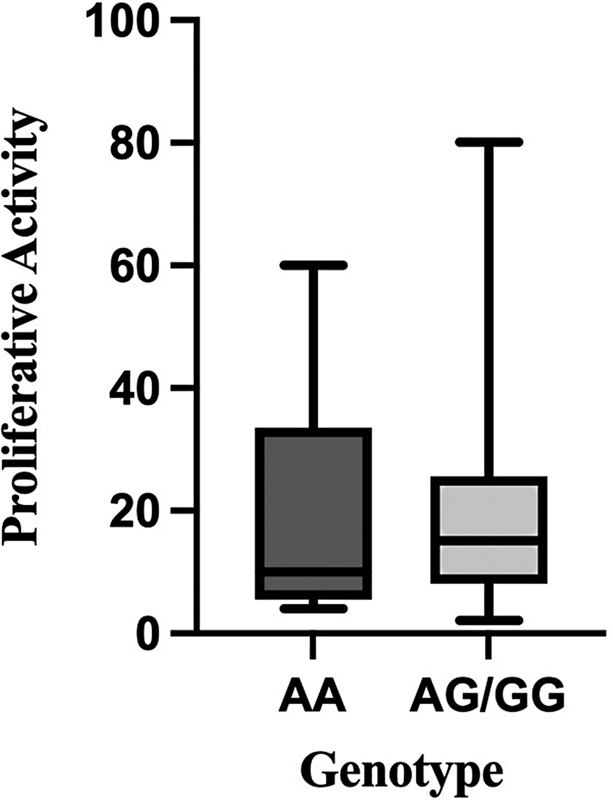
Box and whiskers plot comparing proliferative activity in patients with AA and AG/GG genotype.

Patients' proliferative activity was compared with an unpaired *t* -test between two groups of AA and AA/AG. Mean proliferative activity in patients with AA genotype is 20.77 and in AG/GG is 19.34, and there is no statistical significance between them ( *t* = 0.2575, *p* = 0.7980). Although when data are put into a box and whiskers plot, there are obvious outliers in proliferative activity of patients with AG/GG genotype ( Fig. 2 ).

## Discussion

The IL-10 SNP at -1082 has been associated with cytokine inducibility and expression variation. A nuclear protein physically interacts, in an allele-specific manner, with the promoter region of the IL-10 gene. This protein is poly (ADP-ribose) polymerase 1 (PARP-1). It has been shown that PARP-1 acts as a transcription repressor, with preferential binding for the A-allele than the G-allele. This difference results in less expression in the A allele of this gene. 10

This SNP has been studied in different populations and malignancies, and the results have been conflicting. A study investigated the result of 22 studies in 13 different malignancies regarding the SNPs of the IL-10 gene and found a positive association between IL-10 genotype and disease susceptibility or progression. In some cancers like cutaneous malignant melanoma and prostate cancer, the genotypes that were associated with less expression of IL-10 were a risk factor. However, in other cancers like cervical cancer and hepatocellular cancer, higher expression of IL-10 was a risk factor. The results are especially more conflicting in breast cancer. 11

One study on the Indian population 12 and one on the Italian population 13 found that the low IL-10 expressing AA genotype is associated with higher breast cancer risk.

In this study, the difference we found in the frequency of genotypes between our cases and controls, who were all Georgian women, was not statistically significant. Our findings are in accordance with the finding of two studies on Chinese 14 and Iranian 15 populations.

While these differences might be explained by the dual function of IL-10, as an immunosuppressant (cancer-promoting) and as an antiangiogenic (cancer-inhibiting) agent, it also might be a result of differences in ethnicity.

This study also compared the proliferative activity between cases harboring the G allele with those only having the A allele in IL-10 -1082 SNP for the first time. The proliferative activity was measured by Ki-67 which is a monoclonal antibody that recognizes proliferating cells. 16 And even though there was no statistically significant difference between the mean proliferative activity of these two groups, there are obvious outliers in the group having the G allele with higher proliferative activity shown in Fig. 1 . It is important to note that a higher Ki-67 index is associated with a poorer prognosis and higher recurrence rate in breast cancer patients. 17

## Conclusion

This study showed no real association between -1082 A/G SNP and breast cancer risk in Georgian women. Much larger studies including different ethnicities and studies combining genotype and gene expression are required to improve our understanding of the role of IL-10 in cancer development, as this current study comes with limitations in size and a lack of full phenotype status (for example, the presence of metastasis). Further understanding of IL-10's role in cancer development can aid us in new cytokine immunotherapies in malignancies.
